# A multi-surgeon learning curve analysis of overall and site-specific positive surgical margins after RARP and implications for training

**DOI:** 10.1007/s11701-022-01378-w

**Published:** 2022-02-28

**Authors:** Carlo Gandi, Angelo Totaro, Riccardo Bientinesi, Filippo Marino, Francesco Pierconti, Maurizio Martini, Andrea Russo, Marco Racioppi, PierFrancesco Bassi, Emilio Sacco

**Affiliations:** 1 Department of Urology, Fondazione Policlinico Universitario A. Gemelli IRCCS-Università Cattolica del Sacro Cuore, Rome, Italy; 2 Department of Anatomic Pathology and Histology, Fondazione Policlinico Universitario A. Gemelli IRCCS-Università Cattolica del Sacro Cuore, Rome, Italy; 3 Department of Anesthesiology and Intensive Care, Fondazione Policlinico Universitario A. Gemelli IRCCS-Università Cattolica del Sacro Cuore, Rome, Italy

**Keywords:** Prostate cancer, RARP, Robotic prostatectomy, Positive surgical margin, Learning curve, Training

## Abstract

Robot-assisted radical prostatectomy (RARP) is the most adopted treatment for localized prostate cancer. The aim of this study was to explore the learning curves (LC) for overall and site-specific positive surgical margins (PSM) occurrence after RARP of multiple surgeons within a step-structured mentor-initiated training program. The study included consecutive patients undergoing RARP between January 2013 and March 2020, by three surgeons: a mentor and his two trainees. Prospectively collected patients’ data were retrospectively analyzed. The cumulative summation (CUSUM) method was used to generate the LCs, with turning points indicating the number of cases to reach proficiency levels. Furthermore, the association between PSM and surgical experience was evaluated, adjusting for case mix. A total of 761 consecutive patients were included, 370 treated by the Mentor surgeon, 247 and 144 treated, respectively, by the two Trainees. Mentor and Trainees had similar PSM rates (31.6% vs 28.0% vs 31.3%, *p* = 0.6). CUSUM charts showed different LC shapes for different PSM locations (postero-lateral, bladder neck, apex, and multifocal/> 3 mm). Surgical experience was significantly associated with overall, postero-lateral, and multifocal/> 3 mm PSMs, in the Mentor series only. Trainees reached their turning points after far fewer cases then the Mentor, both for overall (12 and 31 vs 153), postero-lateral (24 and 30 vs 120), and multifocal/> 3 mm PSMs (9 and 31 vs 153). The achievement of stable SM proficiency takes involved different LCs depending on the prostatic location being considered. Monitoring site-specific LC can indicate the surgical steps for which there may be still room for further technical refinements, even when an apparent proficiency status seems achieved.

**Supplementary Information:**

The online version contains supplementary material available at 10.1007/s11701-022-01378-w.

## Introduction

Robot-assisted radical prostatectomy (RARP) is the most adopted treatment of localized prostate cancer [1, 2]. The main goal of oncological surgery is cancer control, which, during radical prostatectomy (RP) for prostate cancer, means the control of surgical margins (SM). Positive SM (PSM) after RP are associated with a greater risk of biochemical recurrence (BCR) and patient anxiety [3, 4]. Furthermore, there are increasing evidences on the impact of location, number, and length of PSM after RP in predicting BCR [5, 6]. As a result, the achievement of proficiency in SM control should be the most important goal in RARP training. A thorough knowledge of surgeons’ learning curve (LC) may be useful for the development of effective and standardized training programs that guarantee the achievement of a baseline expertise, without jeopardizing the patient's safety during the learning phase [7]. Several LC studies have shown the impact of surgical experience on the outcomes of RARP, focusing more often on functional outcomes, operative time, and estimated blood loss [8–10]. Few studies have shown the existence of an LC for PSM occurrence after RARP [11–14], and no studies have shown the existence of different LC for different locations, number, and length of PSM.

The aim of this study was to explore, using the cumulative summation (CUSUM) method, the learning curves for overall and site-specific PSM occurrence after RARP of three surgeons within a step-structured mentor-initiated training program with a dual console system, in a second-generation robotic tertiary referral center.

## Materials and methods

### Patient population, study design, and data assessment

All consecutive patients undergoing RARP, between January 2013 and March 2020, at our institution were included in this IRB-approved study after signing an informed consent. Prospectively collected patients’ data were retrospectively analyzed. PSM locations have been classified into apex, right (RPL), and left (LPL) postero-lateral, bladder neck (BN), according to a multi-institutional study by Patel et al. [15]. Multifocal PSM or PSM longer than 3 mm (MF/> 3 mm) were also recorded [16]. The surgeon performing each surgical step was recorded in the operation report. Overall 42 (5.5%) procedures involved two surgeons (the Mentor and one of the Trainees). Recorded PSMs were attributed to the surgeon responsible for the surgical step that was crucial for the specific PSM location.

### Surgical technique and training program

All procedures were performed following a standardized four-arms RARP procedure (Montsouris technique [17], using a transperitoneal six-port approach, 0° lens, da Vinci SI^®^ (from 2013 to December 2015) or da Vinci XI^®^ (from January 2016) robotic surgical system (Intuitive Surgical Inc.). Briefly, an initial posterior approach to the seminal vesicles from Douglas’ pouch was used; BN preservation was attained whenever technically possible; planned, intrafascial, or interfascial nerve-sparing technique (NS) was performed selectively based on side-specific risk assessment, including multiparametric MRI findings available for most patients in our series; apical dissection was carried out using an anterior approach, after controlling with resorbable stiches and cutting the dorsal venous complex (DVC), and circularly freeing and cutting the urethra a few millimeters away from the apex. Vesico-urethral anastomosis was performed using the Van Velthoven technique.

Surgical procedures have been performed by three right-handed surgeons. The Mentor had a large previous open RP experience and started performing RARP after a 6-month training period, including simulation and observership in qualified robotic surgery centers. Trainee 1 and Trainee 2 had no previous RP experience before starting to perform RARP at our institution through a step-structured mentor-initiated training program.

The training program included a period of didactic sessions regarding robotic mechanics and instrumentations, dry and wet lab. The next step of the program consisted in becoming proficient as bedside assistant of the Mentor, thus learning the steps of the RARP procedure. The program culminated in the trainees performing specific parts of the procedure under supervision of the Mentor, ready to take over thanks to a dual console system. Console training was divided into five steps: (1) dissection of seminal vesicles and posterior prostate, bladder detachment, incision of endopelvic fascia, and dorsal venous complex (DVC) ligation; (2) BN incision, control of prostatic pedicles and NS; (3) DVC transection, apex dissection and transection of the urethra; (4) suturing the vesicourethral anastomosis; (5) pelvic lymphadenectomy. A trainee could move on to the next step when judged proficient by the Mentor.

### Pathological evaluation

RARP specimens were evaluated by two academic uro-pathologists (PF and MM). Each radical prostatectomy specimen was totally embedded and processed with the whole-mount method. Each specimen was weighed, measured, inked, and fixed in 10% neutral formalin. After fixation, the apex and base were amputated and serially sectioned at 4 mm intervals in the vertical para-sagittal plane. The seminal vesicles were sectioned parallel to their junction with the prostate and entirely submitted for evaluation. The remaining specimen was serially sectioned perpendicular to the long axis of the gland from the apex to the base. Whole-mount sections were prepared at 5 mm sections and stained with hematoxylin and eosin for histological evaluation. When immunohistochemical studies were necessary, the paraffin block with the whole-mount section was divided in four microblocks (anterior-lateral left and right quadrants, posterior-lateral left and right quadrants). A PSM was defined as cancer cells seen at the inked margin [18, 19].

### Cumulative sum (CUSUM) method

The CUSUM method was used to generate retrospectively the learning curves for overall and site-specific PSM occurrence. Overall PSM learning curve was also stratified by pathological stage. We chose the most intuitive CUSUM technique, that is the cumulative “observed minus expected failure” method which plots the procedures chronological sequence number on the horizontal axis against the cumulative sum of observed minus expected failure rate on the vertical axis [20, 21].

While there is a plenty of literature regarding the acceptable range of overall PSM rate after RARP [22], there are no such consistent reporting for different PSM locations, number, and length. Because of this gap in the literature, as expected minimum proficiency levels (expected failure rate) for overall and site-specific PSM rate, we chose the PSM rates reached by the Mentor in the last part of his case series, when his PSM rates stabilized at a plateau level, according to the methodology described by Rogers et al. [23]. For each CUSUM curve, we defined a turning point as a peak point after which the PSM rate begins to decrease and then reaches the proficiency level without increasing significantly again.

### Statistical analysis

Mean and standard deviation or median and 95% confidence interval (CI) were used to report continuous variables, as appropriate. Chi-squared test was used to compare categorical variables and ANOVA to compare continuous variables. Univariate and multivariate logistic regression analyses were used to evaluate the association between PSMs and potential predictors for the whole patient population. To evaluate the association between PSM and surgical experience, adjusting for case mix, we built, for each surgeon, a multivariate model regressing the overall and site-specific SM status on surgical experience and the independent predictors of PSM in our series. Surgical experience was defined splitting equally each surgeon’s case series into three groups of consecutive patients according to the chronological order based on procedure date [11].

A two-sided *p* < 0.05 was deemed to indicate statistical significance. Statistical analyses were performed using MedCalc software for Windows v.12.3.0 (MedCalc Software, Mariakerke, Belgium).

## Results

### Patients and PSM characteristics

Table 1 displays peri-operative, intra-operative, and post-operative characteristics of the 761 consecutive included patients. Overall PSM rate was 30.3% (231/761). No significant differences in patient characteristics emerged between the surgeons.

**Table 1:** Pre-operative, intra-operative, and post-operative patient characteristics

	Overall	Surgeon 1	Surgeon 2	Surgeon 3	*p* Value
	(*n* = 761)	(*n* = 370)	(*n* = 247)	(*n* = 144)	
Pre-operative variables					
Age, median (95% CI)	67 (67—68)	67 (66—67)	68 (66—69)	67 (66—69)	0.10
BMI, mean ± SD	26.3 ± 2.9	25.9 ± 2.8	26.6 ± 3.1	26.6 ± 2.6	0.50
Prostate volume ml, mean ± SD	42.6 ± 19.9	43.1 ± 21.1	41.5 ± 17.9	43.1 ± 19.9	0.57
PSA ng/ml, mean ± SD	8.9 ± 6.8	8.9 ± 6.7	8.9 ± 7.6	9.1 ± 5.4	0.99
Biopsy ISUP grade, *n* (%)					
1–2	537 (70.5)	266 (71.9)	175 (70.9)	96 (66.7)	0.44
3	123 (16.2)	49 (13.2)	44 (17.8)	30 (20.1)	0.08
4–5	101 (13.3)	55 (14.9)	28 (11.3)	18 (12.5)	0.43
Clinical stage, *n* (%)					
cT1	508 (66.7)	247 (66.7)	169 (68.4)	92 (63.9)	0.66
cT2	253 (33.3)	123 (33.3)	78 (31.6)	52 (36.1)	0.65
Intraoperative variables					
Nerve-sparing technique, *n* (%)	325 (42.7)	147 (39.7)	103 (41.7)	71 (49.3)	0.06
Postoperative variables					
Pathologic staging, *n* (%)					
pT2	571 (75.1)	279 (75.4)	187 (75.3)	105 (72.9)	0.80
pT3	190 (24.9)	91 (24.6)	60 (24.7)	39 (27.1)	0.81
Pathologic ISUP grade, *n* (%)					
1–2	535 (70.3)	270 (72.9)	169 (68.4)	96 (66.7)	0.27
3	172 (22.6)	69 (18.7)	64 (25.9)	39 (27.1)	0.04
4–5	54 (7.1)	31 (8.4)	14 (5.7)	9 (6.2)	0.39
Percentage of tumor, mean ± SD	6.2 ± 8.7	5.8 ± 7.1	6.5 ± 9.1	6.5 ± 10.9	0.48
Perineural invasion, *n* (%)	536 (70.4)	248 (67.0)	180 (72.8)	108 (86.8)	0.12
PSM, *n* (%)	231 (30.3)	117 (31.6)	69 (27.9)	45 (31.3)	0.6

*BMI* body mass index, *PSA* prostate-specific antigen, *ISUP* International Society of Urological Pathology, *PSM* positive surgical margin

Table 2 shows SM status by location/extension and surgeon. The apex was the most common site of PSM (108/761, 14.3%), followed by the postero-lateral location (82/761, 10.8%) and the bladder neck (69/761, 9.1%). Within the postero-lateral location, a significantly higher PSM rate was observed for the left side (RPL/LPL: 4.5%/7.0%, *p* = 0.04).

**Table 2:** Surgical margin status by location/extension and surgeon

	Overall	Mentor	Trainee 1	Trainee 2	*p* Value
	*n* = 761 (100%)	*n* = 370 (100%)	*n* = 247 (100%)	*n* = 144 (100%)	
PSM	227 (29.8)	117 (31.6)	69 (28.0)	45 (31.3)	0.6
Apex	108 (14.2)	55 (14.9)	28 (11.3)	26 (18.0)	0.17
Bladder neck	69 (9.1)	29 (7.8)	29 (11.7)	11 (7.6)	0.2
Postero-lateral	82 (10.8)	43 (11.6)	19 (7.7)	20 (13.8)	0.12
Right-PL	34 (4.5)	17 (4.5)	9 (3.6)	8 (5.5)	0.66
Left-PL	53 (7.0)	27 (7.3)	12 (4.8)	14 (9.7)	0.17
Multifocal or > 3 mm	140 (18.4)	78 (21.0)	36 (14.6)	26 (18.1)	0.12

There may have been more than one positive margin location for each patient with PSM

*PSM* positive surgical margin, *PL* postero-lateral

The PSM rate for pT2 and pT3 cases was 23.3% (133/570) and 50.8% (97/191), respectively (*p* < 0.0001). The pT3 PSM rate was significantly higher than pT2 PSM rate even for each specific PSM location (see the table in Online Resource 1). No statistically significant differences were found between the surgeons for the incidence of PSM stratified by location/extension.

### CUSUM analysis

**Fig. 1:**
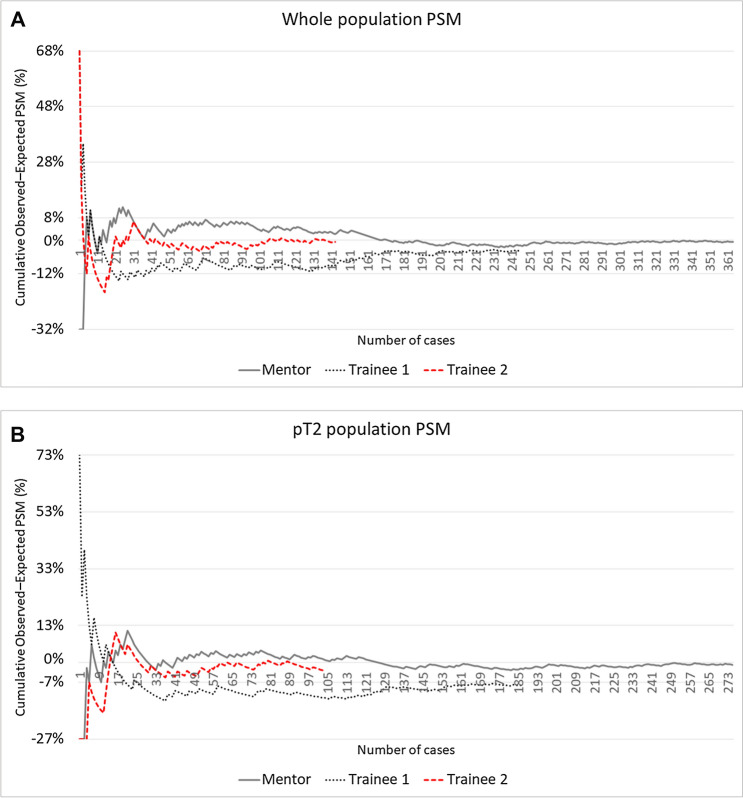
CUSUM charts with learning curves for overall PSM occurrence of each surgeon in the whole population (**A**) and in the pT2 population (**B**)

Figure 1 shows CUSUM charts with learning curves for PSM occurrence in the whole population (Fig. 1A) and in the pT2 population (Fig. 1B) of each surgeon. A prominent reduction in the PSM rate, indicating improving performance, was evident for all surgeons and in both patient populations. For each surgeon, it was possible to identify a three-phase learning curve: an initial upward trend (phase 1), a phase of decline culminating with a turning point (phase 2) after which the PSM rate decreases to or below the proficiency level, reaching the plateau (phase 3) in the overall population. Mentor's turning point (case 153) was set much further in his overall case series than that of Trainee 1 (case 12) and Trainee 2 (case 31). The same trend, for each surgeon, was noted in the pT2 population chart (Mentor’s turning point at 118 cases; Trainee 1 at 14 cases; Trainee 2 at 21 cases).

**Fig. 2:**
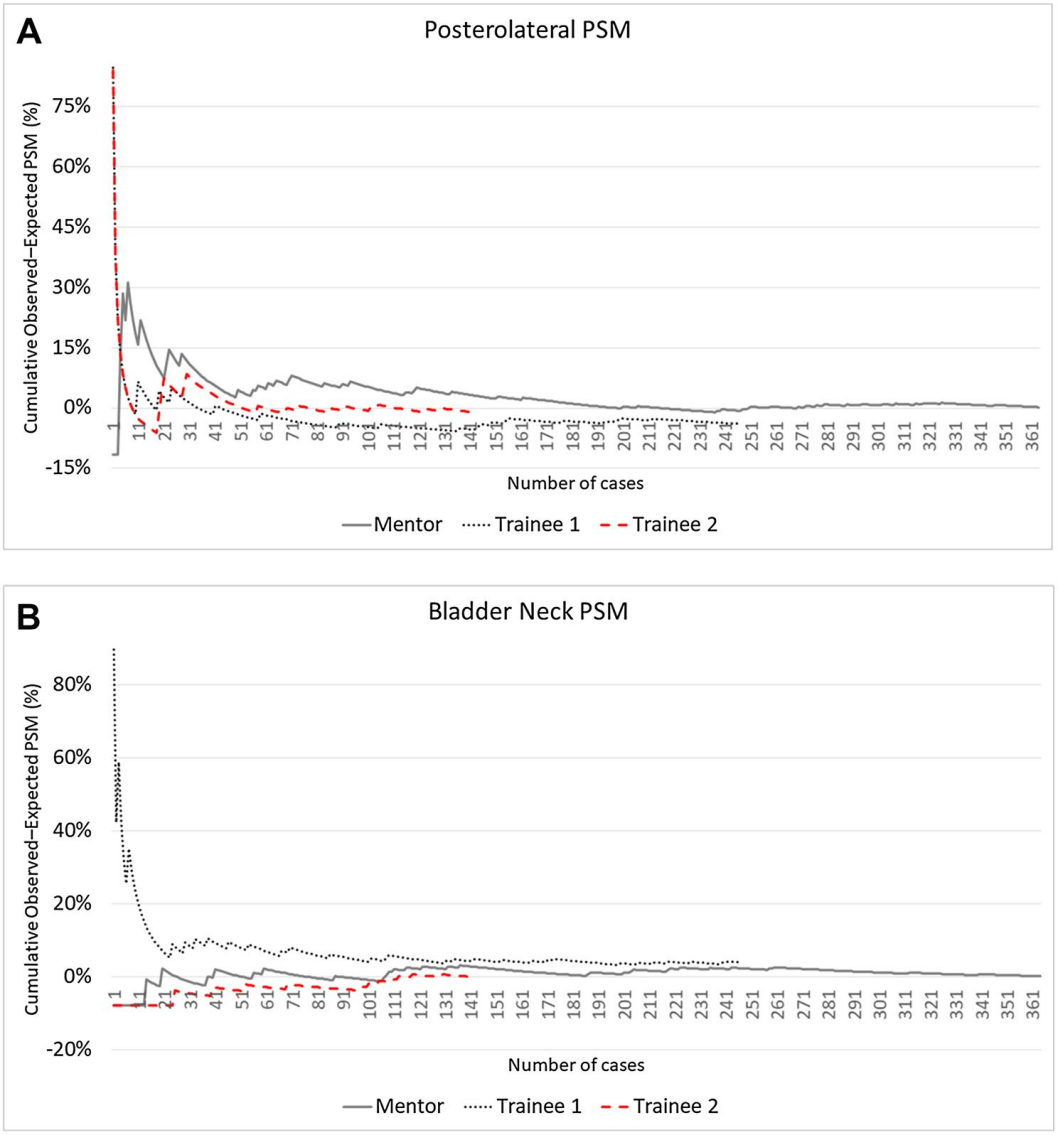

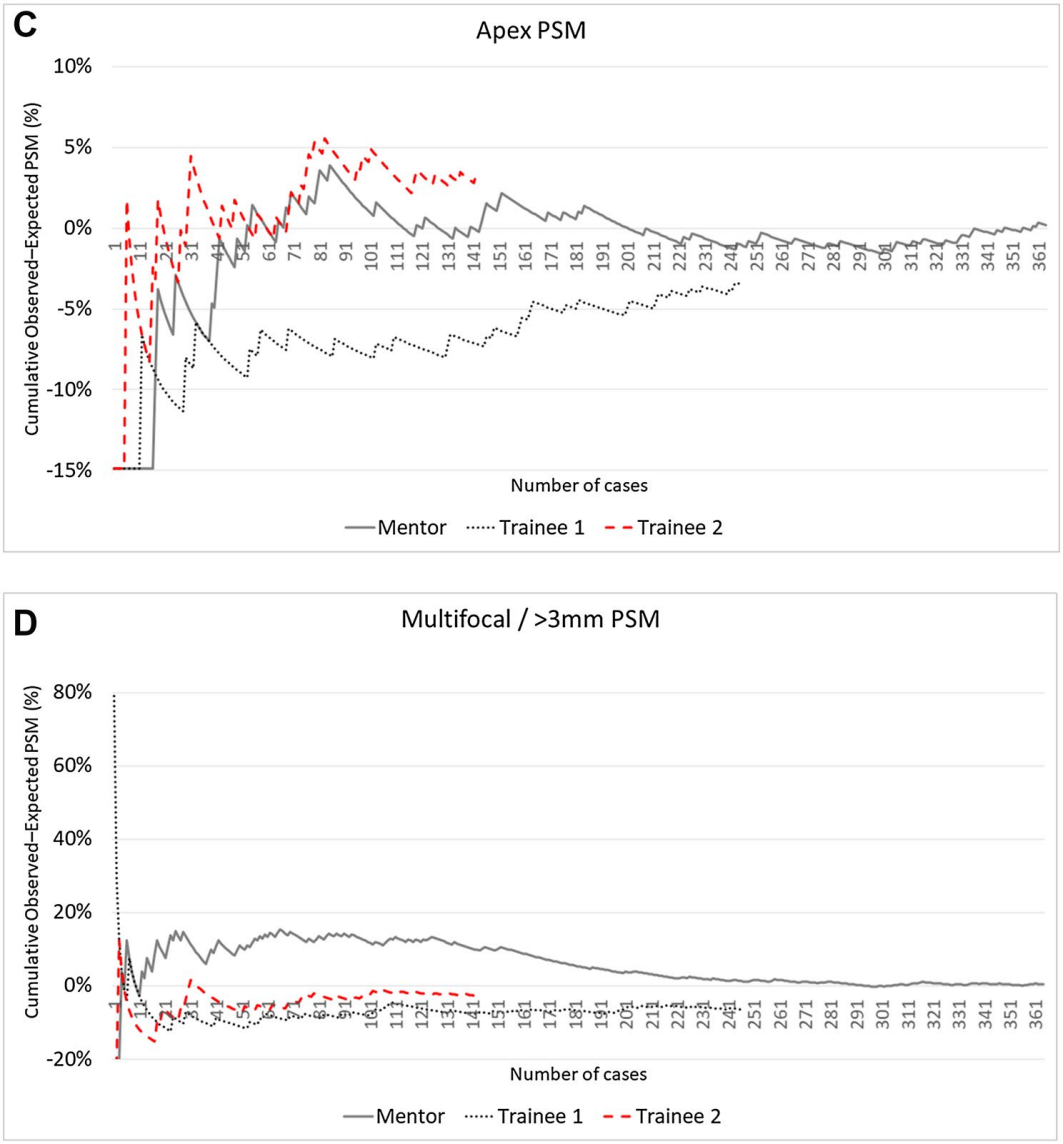
CUSUM charts with learning curves for PSM occurrence of each surgeon for postero-lateral location (**A**), bladder neck (**B**), apex (**C**), and multifocal/> 3 mm PSM (**D**) in the whole population

Figure 2 shows CUSUM charts of the three surgeons for the different PSM locations and for multifocal or > 3 mm PSM in the whole patient population.

The CUSUM_POSTEROLATERAL_ chart (Fig. 2A) showed a clear learning curve effect for all the three surgeons, with the recognizable three phases: again, the decline phase was longer and more gradual for the Mentor (turning point at case 120), shorter for Trainee 1 and Trainee 2 (turning points at case 24 and case 30, respectively); the final plateau phase set around a common 10% rate.

The CUSUM_BLADDER-NECK_ curves (Fig. 2B) identified, for all three surgeons, the achievement of a plateau at a 10% rate after about 100 cases. The very early phase of the curve was characterized by inhomogeneous trends: while for Trainee 1, there was a learning curve effect characterized by a progressive reduction in the PSM rate as the surgeon’s experience increased, for Mentor and Trainee 2, we registered a progressive stabilization after a fluctuating trend in a narrow range (5–15%).

Looking at the CUSUM_APEX_ chart (Fig. 2C), a learning curve effect cannot be identified for any of the surgeons, with the PSM rate fluctuating in a wide range around 15%, without an identifiable trend toward a plateau.

The three-phase learning curve can also be identified in the CUSUM_MF or >3 mm_ chart (Fig. 2D), with a trend that imitates, for each surgeon, that of the general PSM learning curve: even in this case, the turning points of Trainee 1 (case 9) and Trainee 2 (case 31) occurred earlier than that of the Mentor (case 153).

### Multivariate analysis

Univariate and multivariate logistic regressions for PSM predictors performed for the whole patient population are displayed in Table 3. Prostate volume, preoperative PSA, nerve-sparing technique, pathological stage, perineural invasion, and percentage of cancer in the surgical specimen were statistically significant independent predictors for PSM occurrence.

**Table 3:** Univariate and multivariate analyses for PSM predictors (whole population)

	Univariate analysis			Multivariate analysis		
	OR	95% CI	*p* Value	OR	95% CI	*p* Value
Pre-operative variables						
Age (continuous)	1.02	0.99—1.04	0.07			
BMI (continuous)	0.99	0.99—1.00	0.71			
Prostate volume (continuous)	0.98	0.98—0.99	0.02*	0.98	0.98—0.99	0.01*
PSA ng/ml (continuous)	1.03	1.01—1.05	0.01*	1.02	1.01—1.05	0.02*
Clinical stage T2 vs T1	1.19	0.86—1.66	0.28			
Intraoperative variables						
Nerve-sparing technique	1.12	1.07—1.21	0.02*	1.56	1.07—2.27	0.01*
Postoperative variables						
Pathological stage T3 vs T2	3.4	2.40—4.80	< 0.0001*	1.90	1.25—2.88	0.002*
Pathological ISUP grade						
ISUP 3 vs 1–2	2.33	1.62—3.34	< 0.0001*	1.31	0.85—2.02	0.21
ISUP 4–5 vs 1–2	2.90	1.64—5.13	0.0002*	1.63	0.82—3.21	0.15
Percentage of cancer (continuous)	1.1	1.09—1.12	< 0.0001*	1.06	1.03—1.09	< 0.0001*
Perineural invasion	4.52	2.84—7.17	< 0.0001*	2.58	1.55—4.28	0.0002*

*Statistically significant

*PSM* positive surgical margin, *BMI* body mass index, *PSA* prostate-specific antigen, *ISUP* International Society of Urological Pathology

Table 4 shows the multivariate models assessing the association between surgical experience and overall and site-specific PSM occurrences adjusted for statistically significant independent predictors of PSM, for each of the surgeons. The Mentor’s model revealed a significant association between surgical experience and overall, PL and MF or > 3 mm PSM, while Trainees’ models revealed not such associations.

**Table 4:** Multivariate analysis by PSM location/extension for the three surgeons: mentor (A), trainee 1 (B) and trainee 2 (C)

	Overall PSM		Postero-lateral PSM		Apex PSM		Bladder neck PSM		MF or > 3 mm PSM	
	OR	*p* Value	OR	*p* Value	OR	*p* Value	OR	*p* Value	OR	*p* Value
(A) Mentor										
Prostate volume (continuous)	0.99	0.79	1.01	0.41	1.01	0.85	0.98	0.26	1.00	0.59
PSA ng/ml (continuous)	0.99	0.76	1.04	0.20	0.90	0.01*	0.99	0.79	1.06	0.03
Nerve-sparing technique	1.55	0.10	1.35	0.49	1.68	0.15	0.88	0.82	1.3	0.46
Pathological stage T3 vs T2	1.38	0.31	3.14	0.008*	1.28	0.53	1.2	0.83	1.32	0.48
Percentage of cancer (continuous)	1.1	0.0002*	1.05	0.1	1.1	0.002*	1.1	0.002*	1.21	< 0.0001*
Perineural invasion	3.12	0.001*	1.59	0.38	3.74	0.004*	1.84	0.32	3.00	0.01*
Surgical experience (patient groups)										
Group 2 vs 1	0.38	0.004*	0.26	0.007*	0.64	0.29	0.69	0.46	0.12	< 0.0001*
Group 3 vs 1	0.46	0.02*	0.43	0.06*	0.73	0.44	0.15	0.05	0.24	0.0003*
(B) Trainee 1										
Prostate volume (continuous)	1.01	0.65	0.97	0.24	1.01	0.39	1.02	0.15	1.00	0.63
PSA ng/ml (continuous)	1.02	0.33	1.00	0.92	0.98	0.62	1.05	0.12	1.07	0.03*
Nerve-sparing technique	2.11	0.06	2.06	0.28	2.42	0.08	1.73	0.32	2.12	0.16
Pathological stage T3 vs T2	2.84	0.01*	2.23	0.18	1.03	0.94	2.84	0.06	0.71	0.53
Percentage of cancer (continuous)	1.11	0.0005*	1.01	0.89	1.05	0.08	1.06	0.005*	1.06	0.005*
Perineural invasion	11.98	0.001*	88.0	0.99	11.57	0.02	10.18	0.03*	125.0	0.99
Surgical experience (patient groups)										
Group 2 vs 1	0.94	0.89	1.89	0.38	0.98	0.97	0.42	0.15	0.94	0.64
Group 3 vs 1	0.93	0.87	1.38	0.66	1.58	0.41	0.41	0.11	0.92	0.16
(C) Trainee 2										
Prostate Volume (continuous)	1.00	0.81	1.01	0.47	1.01	0.53	0.98	0.41	0.98	0.36
PSA ng/ml (continuous)	1.03	0.40	1.07	0.12	0.99	0.87	1.03	0.69	1.06	0.14
Nerve-sparing technique	1.61	0.10	2.78	0.14	1.67	0.40	0.52	0.44	1.07	0.90
Pathological stage T3 vs T2	2.28	0.04*	1.66	0.37	1.20	0.73	1.28	0.74	1.04	0.93
Percentage of cancer (continuous)	1.08	0.001*	1.01	0.74	1.06	0.03	1.03	0.14	1.03	0.05
Perineural invasion	4.61	0.003*	38.0	0.99	44.9	0.99	8.7	0.99	44.6	0.99
Surgical experience (patient groups)										
Group 2 vs 1	0.98	0.97	0.99	0.84	1.51	0.46	1.02	0.97	1.28	0.22
Group 3 vs 1	0.85	0.74	0.98	0.99	0.57	0.38	2.84	0.24	1.02	0.68

*Statistically significant

*PSM* positive surgical margin, *PSA* prostate-specific antigen

## Discussion

As far as we know, this is the first study to show the existence of different learning curves for different PSM locations in RARP. Two previous studies have shown that the CUSUM method is a useful tool for evaluating PSM learning curve of RARP, but they did not evaluate the site-specific PSM occurrence [12, 13]. Williams et al. monitored PSM rate during a single surgeon transition from open to robotic RP in a series of 158 patients with pT2 disease: using CUSUM method, they found a turning point at procedure 50 (PSM rate 22%) with a subsequent flattening trend (PSM rate 13%) after 110 procedures [12]. Sivaraman et al. used CUSUM method to analyze their institutional RARP series (initiated after 250 laparoscopic RP) as a whole (9 surgeons), finding a turning point after 100 procedures (PSM rate 20%) [13].

Within our multi-surgeon learning curve analysis, we documented a statistically significant association between surgical experience and overall, PL and MF/> 3 mm PSM occurrences, but only for the Mentor surgeon and not for the Trainees. We interpreted these results based on the analysis of the CUSUM charts, which showed Trainees reaching proficiency in SM control much earlier than their Mentor. Reaching their turning points after far fewer cases than their Mentor, Trainees proved to benefit significantly from institutional experience built by the Mentor who imported the surgical technique and standardized it within the hospital. In line with our results, Schroeck et al. (2008) showed how the trainees of an experienced mentor had, in the early phase of a mentor-initiated RARP training program, lower PSM rates than their mentor in the same phase [24], although site-specific PSM rates were not assessed.

In our study, we identified different CUSUM curve shapes for different PSM locations, with implications for the different steps of RARP training.

The CUSUM_POSTEROLATERAL_ curves showed a clear learning curve effect, shaping predominantly the early phase of the overall PSM learning curve, with Trainees reaching their turning points earlier than their Mentor. In our series, NS technique proved to be an independent risk factor for PSM; therefore, a faster mastery of NS technique by the Trainees may be an explanation for the anticipation of their turning points. Other authors reported an association between NS and increased risk of PSMs [25–27], even though the issue remains controversial [28].

For the postero-lateral location, we also documented a statistically significant difference in the PSM rate between the right and left side, with the left postero-lateral being associated with a higher risk of PSMs. The left-sided dominance of PSM has been previously described by other authors, both in robotic [29] and laparoscopic RP [30]. An explanation may be the asymmetry in the mutual position of robotic instruments (scissors and forceps) and the different prostatic side, introducing inherent technical limits during the left postero-lateral dissection for right-handed surgeons. More conflicts between bipolar and Prograsp forceps, limitations in the range of motion of the fourth arm, and right robotic instruments reaching the left postero-lateral aspect of the gland in a somewhat uncomfortable way, make more technically challenging the left postero-lateral dissection. In terms of training, we believe that PL dissection, even with NS intent, can be safely introduced in the early steps of learning if under the supervision of a skilled mentor, paying greater attention to the dissection of the side opposite the surgeon's dominant hand.

The CUSUM_BLADDER-NECK_ chart showed inhomogeneous trends between the three surgeons in the very early phase of the experience (first 20 cases), and then a phase of transition with fluctuating PSM rates in a narrow range (5–15%) before reaching a common plateau phase at 10% rate (after about 100 cases, for all the surgeon). A possible interpretation for the fluctuation phase may be the progressive acquisition of mastery of the bladder neck sparing technique (BNS), but we had not enough data on the BN dissection technique applied for all the patients, to test this hypothesis. BNS can be problematic due to the junction’s anatomical variability and lack of natural visual land-marks [31]. However, there is no consensus in the literature on BNS as a risk factor for PSM, as highlighted in a recent review on the topic by Bellangino et al. [32]. The same lack of consensus concerns the association between BNS and BCR: in a large RARP series of > 1000 patients, Friedlander et al. reported no difference in BCR-free survival for BNS and non-sparing groups [33]. According to our experience BNS can be safely introduced in the early phases of a mentor-initiated training, avoiding, at the beginning, cases with large prostate, prominent median lobes and known disease at the base of the gland.

The apex was the most common PSM location in agreement with several robotic [15, 29, 34], laparoscopic [29, 35], and open [29, 34] radical prostatectomy series. CUSUM_APEX_ chart did not show a peculiar curve for this site, nor a significant reduction in the PSM rate associated with improving surgical experience, not even for the Mentor with the most extensive series. This reflects the challenging nature of apex dissection, where maximizing urethral length and sparing neurovascular bundles can jeopardize complete tumor resection balancing [36]. Other reasons for the high apex PSM rate are its variable configuration and the poor representation of capsule and peri-prostatic tissues in this location, which determine more frequent iatrogenic intra-prostatic incisions and a more difficult distinction between intra-prostatic and extra-prostatic tissue for the pathologist, all leading to artifacts or "false" PSMs [3, 37]. A study by Marcq et al. found that only extensive apical PSMs, but not focal apical PSMs, were independently associated with biochemical recurrence (BCR) in their series; as an explanation of their results, they cited the greater presence of artifacts among the focal PSMs [38]. For all these reasons, we think that apex dissection, for its demand of precision, still must be part of the advanced steps of training programs, but always keeping in mind that surgeon experience is only one of the many variables determining apex PSMs.

Avoiding multifocal PSM and PSM length > 3 mm is an outcome to be achieved as important as avoiding the presence of PSM itself, for its recognized impact on the risk of BCR [4, 5, 15]. In our study, the learning curves identified in the CUSUM_MF or >3 mm_ chart showed trends imitating, for each surgeon, that of the general PSM learning curve, confirming the great advantage of the trainees who reached the institutional plateau threshold after much less cases of the Mentor who set that threshold.

This study has its strength in the large contemporary series of consecutive patients in a high-volume center, in the multiple surgeons evaluation based on two effective tools for learning curve assessment (CUSUM analysis and multivariate logistic regression to adjust for case mix) [20] and in the standardized protocol for assessing the prostatectomy specimens by two experienced uro-pathologists. However, we acknowledge several limitations, including the retrospective nature, the lack of data on BCR to evaluate its association with PSMs, and the lack of enough data on BNS technique to test it as a PSM predictor. Another limitation could be the single-center design, though it is difficult and unreliable to compare different surgeons from different institutions in terms of PSM rate, considering that institutions differ, from each other, in many aspects such as patient characteristics, surgical technique, and post-operative pathological evaluation. Moreover, even though CUSUM curves are recognized effective tools for the continuous assessment of surgeons’ progress, it is difficult to compare curves of different surgeons, because LC must be appraised as an individual entity, depending on several factors that may influence the characteristics of an individual learning curve, such as surgeon experience, skills, and innate characteristics, and continuous refinements in personal surgical technique. We also acknowledge that our overall and pT2 PSM rates fell close to the upper limit of the range in the literature (6.5–32% and 4–23% [38]), although several factors may explain differences in PSM occurrence between different institutions.

## Conclusions

The achievement of stable SM proficiency takes different caseloads with distinct learning curves depending not only on cancer features but also on the institutional experience and the prostatic location being considered. A step-structured mentor-initiated RARP training program can reduce trainees’ PSM rate and extension within acceptable limits in the early phase of training, thus without jeopardizing patient's oncological safety. This study supports the CUSUM method as an effective tool for surgeon self-appraisal to prompt continuous quality improvement. Monitoring site-specific learning curves can indicate the surgical steps for which there may be still room for further technical refinements, even when an apparent proficiency status seems to have been achieved.

## Supplementary Information

Below is the link to the electronic supplementary material.

Supplementary file1 (XLSX 12 KB)

## Data Availability

Data related to patients and surgical procedures can be found at the Department of Urology, Fondazione Policlinico Universitario A. Gemelli IRCCS-Università Cattolica del Sacro Cuore, Largo Agostino Gemelli 8, 00168 Rome, Italy. The results of histopathologic exams are available at the Department of Anatomic Pathology and Histology, Fondazione Policlinico Universitario A. Gemelli IRCCS-Università Cattolica del Sacro Cuore, Largo Agostino Gemelli 8, 00168 Rome, Italy.
